# Relationship between intraoperative dopamine infusion and postoperative acute kidney injury in patients undergoing open abdominal aorta aneurysm repair

**DOI:** 10.1186/s12871-022-01624-6

**Published:** 2022-03-26

**Authors:** Seohee Lee, Dongnyeok Park, Jae-Woo Ju, Jinyoung Bae, Youn Joung Cho, Karam Nam, Yunseok Jeon

**Affiliations:** Department of Anesthesiology and Pain Medicine, Seoul National University Hospital, Seoul National University College of Medicine, 101 Daehak-ro, Jongno-gu, Seoul, Republic of Korea 03080

**Keywords:** Abdominal aortic aneurysm repair, Acute kidney injury, Dopamine, Postoperative complication, Norepinephrine

## Abstract

**Background:**

Acute kidney injury (AKI) is one of the most common complications in patients undergoing open abdominal aortic aneurysm (AAA) repair. Dopamine has been frequently used in these patients to prevent AKI. We aimed to clarify the relationship between intraoperative dopamine infusion and postoperative AKI in patients undergoing open AAA repair.

**Methods:**

We analyzed 294 patients who underwent open AAA repair at a single tertiary center from 2009 to 2018, retrospectively. The primary outcome was the incidence of postoperative AKI, determined by the Kidney Disease Improving Global Outcomes definition, after open AAA repair. Secondary outcomes included survival outcome, hospital and intensive care unit length of stay, and postoperative renal replacement therapy (RRT).

**Results:**

Postoperative AKI occurred in 21.8% (64 out of 294 patients) The risk of postoperative AKI by intraoperative dopamine infusion was greater after adjusting for risk factors (odds ratio [OR] 2.56; 95% confidence interval [CI], 1.09–5.89; *P* = 0.028) and after propensity score matching (OR 3.22; 95% CI 1.12–9.24; *P* = 0.030). On the contrary, intraoperative norepinephrine use was not associated with postoperative AKI (use vs. no use; 19.3 vs. 22.4%; *P* = 0.615). Patients who used dopamine showed higher requirement for postoperative RRT (6.8 vs. 1.2%; *P* = 0.045) and longer hospital length of stay (18 vs. 16 days, *P* = 0.024).

**Conclusions:**

Intraoperative dopamine infusion was associated with more frequent postoperative AKI, postoperative RRT, and longer hospital length of stay in patients undergoing AAA repair, when compared to norepinephrine. Further prospective randomized clinical trial may be necessary for this topic.

**Supplementary Information:**

The online version contains supplementary material available at 10.1186/s12871-022-01624-6.

## Background

Acute kidney injury (AKI) is a serious postoperative complication in surgical patients, [1, 2] leading to an increased length of stay, hospital costs, morbidity, and mortality [3]. Even a mild increase in serum creatinine (SCr) has been reported to be closely related to mortality [4]. AKI is one of the most common complications in patients undergoing abdominal aortic aneurysm (AAA) repair [3, 5, 6]. The incidence of AKI after AAA repair has been reported to be as high as 33% depending on the definition of AKI [5–9] and previous studies have investigated risk factors for AKI development after AAA repair [5, 10–12].

Some previous reports have promoted the use of dopamine infusion to prevent postoperative AKI [13, 14]. Dopamine infusion has been proposed to enhance diuresis and reduce the need for dialysis in patients with postoperative acute renal failure [13]. It has also been reported that a perioperative low-dose dopamine infusion may be a reasonable strategy to reduce postoperative AKI in patients undergoing AAA repair, assuming its effect on increasing renal arterial flow [14]. However, the reno-protective effect of dopamine in various clinical settings is controversial [15, 16]. Furthermore, an intravenous dopamine infusion may worsen renal flow perfusion when used in patients with impaired kidneys [17].

The current study aimed to determine if intraoperative continuous dopamine infusion is associated with the incidence of AKI as defined by the Kidney Disease: Improving Global Outcomes (KDIGO) criteria [18] in patients undergoing open AAA repair. This study also evaluated the relationship between intraoperative dopamine infusion and postoperative outcomes, including survival outcome, after open AAA repair.

## Methods

This was a retrospective observational study, and the study protocol was approved by the Institutional Review Board (IRB) of Seoul National University Hospital (approval no. 1908–091-1055) on 23 August 2019. We performed this retrospective study in compliance with the Declaration of Helsinki and the guidelines for Good Clinical Practice. Written informed consent was waived by the IRB due to the retrospective nature of the study. The data were retrieved from electronic medical records, i.e. form the Seoul National University Hospital Patients Research Environment (SUPREME) system. Postoperative survival data were obtained from the administrative database of death certificates of the National Population Registry of the Korean National Statistical Office. Follow-up was censored on December 31, 2019.

### Study population

All adult patients (≥18 years) who underwent open AAA repair at a single tertiary center (Seoul National University Hospital, Seoul, South Korea) from January 1, 2009, to December 31, 2018, were included in the study. Patients who underwent AAA repair were selected using the hospital procedural code for “abdominal aortic aneurysm repair” and patients who underwent any combined surgery or intraoperative cardiac arrest were excluded. No patient received multiple abdominal aorta surgeries. Patients with a history of chronic kidney disease, end-stage renal disease, renal replacement therapy (RRT), or kidney transplantation were excluded. Other related renal diseases that may affect the postoperative outcome, including congenital single kidney, renal artery stenosis, and atrophied kidney, were also excluded. All patients had baseline SCr and postoperative SCr measurements within 7 days after surgery (median of five measurements). The baseline SCr level was defined as the most recent value measured within 1 month before surgery.

### Data collection

Demographic data, medical history, medication history, preoperative laboratory data, surgical variables, intraoperative variables, and postoperative outcomes were retrospectively collected from electronic medical records (Tables 1 and 2). The anesthetic records of all eligible patients were reviewed to collect intraoperative data, including the use of intraoperative dopamine and norepinephrine infusion. The dopamine or norepinephrine infusion was initiated according to each physician’s decisions when the hypotensive events occurred during an operation in AAA repair.

**Table 1:** Baseline patient characteristics

	Dopamine infusion		*p*-value
	No (*n* = 250)	Yes (*n* = 44)	
Demographic data			
Age (years)	70.8 (9.6)	72.4 (9.5)	0.319
Female	46 (18.4%)	7 (15.9%)	0.854
Body mass index (kg/m^2^)			0.335
< 18.5 kg/m^2^	17 (6.8%)	3 (6.8%)	
18.5–24.9 kg/m^2^	145 (58.0%)	30 (68.2%)	
25.0–29.9 kg/m^2^	74 (29.6%)	11 (25.0%)	
≥ 30.0 kg/m^2^	14 (5.6%)	0 (0.0%)	
Past medical history			
Hypertension	169 (67.6%)	28 (63.6%)	0.733
Diabetes mellitus	35 (14.0%)	6 (13.6%)	1.000
Dyslipidemia	81 (32.4%)	13 (29.5%)	0.842
Cerebrovascular disease	18 (7.2%)	2 (4.5%)	0.749
Angina pectoris	48 (19.2%)	7 (15.9%)	0.759
Myocardial infarction	17 (6.8%)	5 (11.4%)	0.453
Atrial fibrillation	8 (3.2%)	1 (2.3%)	1.000
Chronic obstructive lung disease	18 (7.2%)	4 (9.1%)	0.897
Medication history			
Aspirin	57 (22.8%)	8 (18.2%)	0.629
Clopidogrel	43 (17.2%)	5 (11.4%)	0.456
ACEi/ARB	93 (37.2%)	11 (25.0%)	0.165
ß blocker	66 (26.4%)	14 (31.8%)	0.575
Calcium channel blocker	91 (36.4%)	19 (43.2%)	0.491
Diuretics	36 (14.4%)	6 (13.6%)	1.000
Statin	111 (44.4%)	15 (34.1%)	0.267
Oral hypoglycemic agents	26 (10.4%)	4 (9.1%)	1.000

Data are presented as mean (SD) or number (%)

*ACEi* Angiotensin converting enzyme inhibitor, *ARB* Angiotensin II receptor blocker

**Table 2:** Preoperative data and intraoperative profile

	Dopamine infusion		*p*-value
	No (*n =* 250)	Yes (*n =* 44)	
Preoperative laboratory data			
Serum creatinine (mg/dl)	1.01 (0.3)	1.10 (0.3)	0.052
Hematocrit (%)	39.02 (5.1)	38.07 (6.0)	0.271
Maximal diameter (mm)	59.47 (15.20)	67.64 (17.55)	0.001
Operative data			
Lowest MAP (mmHg)	54 (50–58)	52 (46–59)	0.112
Emergency surgery	28 (11.2%)	9 (20.5%)	0.144
Duration of surgery (min)	350.0 [306.3–408.8]	372.5 [320.0–452.5]	0.199
Supra-renal aneurysm	21 (8.4%)	2 (4.5%)	0.566
Nitroglycerin use	51 (20.4%)	7 (15.9)	0.628
Norepinephrine use	46 (18.4%)	11 (25.0)	0.415
Mannitol use	49 (19.6%)	16 (36.4)	0.023
Furosemide use	80 (32.0%)	26 (59.1)	0.001
Hydroxyethyl starch (l)	0.5 [0–1.2]	1 [0.5–1.5]	0.045
RBC transfusion (units)	1 [0–3]	3 [2–6]	< 0.001

Data are presented as mean (SD), median [IQR], or number (%).

*MAP* Mean arterial blood pressure*, RBC* Red blood cell

### Study outcomes

The primary outcome was the incidence of postoperative AKI during the first 7 days after open AAA repair surgery. The occurrence of AKI was determined by the KDIGO definition [18]. The urine output criterion was not used due to inaccurate or missing data. Secondary outcomes were postoperative RRT, intensive care unit (ICU) length of stay, hospital length of stay, and postoperative survival outcome.

### Statistical analysis

Continuous variables are expressed as the mean (standard deviation) or median (interquartile range) and were compared using a one-way analysis of variance or the Kruskal-Wallis test. Categorical variables are presented as counts (%) and compared using the χ^2^ test. All statistical analyses were carried out using R software (ver. 3.6.1; R Development Core Team, Vienna, Austria). A *p*-value < 0.05 was considered significant.

The primary outcome was analyzed using logistic regression analysis. First, univariable logistic regression analyses were performed with the patient demographics, medical history, medication history, preoperative laboratory data, surgical variables, and intraoperative variables listed in Table 1. Body mass index was categorized according to the World Health Organization’s classification system [19]. Administration of an intraoperative red blood cell transfusion or hydroxyethyl starch was analyzed as a binary variable.

Following univariable analyses, variables with a *p*-value < 0.2 or clinically significant variables were included in multivariable logistic regression analyses without a stepwise variable selection method, resulting in two adjusted models. In model 1, we adjusted for preoperative factors such as demographics, medical history, medication history, and preoperative data. In model 2, we further adjusted for all variables used in model 1 as well as intraoperative variables, including surgical and anesthetic factors.

We performed a propensity score matching analysis to match patients who did and did not receive an intraoperative dopamine infusion using demographic data, past medical history, medication history, preoperative data. Oral hypoglycemic agent was also excluded because of multi-collinearity. In the matching procedure, the nearest neighbor-matching method with 1:3 pairing was used without the caliper because of the reduced number of data. After matching, the incidence of postoperative AKI was compared between the study groups using the χ^2^ test. Seven unbalanced contributors were present with a standardized mean difference > 0.1 after matching, and these variables were used as covariates in the generalized estimating equation (GEE) after propensity score matching. When analyzing matched data by GEE, exchangeable working covariance matrix was used as covariance matrix within matched pairs. In addition, norepinephrine infusion was analyzed in the same way as the analysis for the dopamine infusion. Both the dopamine group and norepinephrine group included patients who used both drugs (11 of 294). The χ^2^ test with Bonferroni correction was performed to determine the incidence of AKI according to the dose of dopamine infusion: renal-dose dopamine infusion (≤ 3 mcg/kg/min) group, more than renal-dose dopamine infusion (> 3 mcg/kg/min) group and no infusion group. The patients missing the dose of dopamine infusion were not included in the analysis of the incidence of AKI according to the dose of dopamine infusion (*n* = 6). The χ^2^ test was performed to determine the incidence of AKI according to the infused drugs: norepinephrine-only infusion group, dopamine-only infusion group, no infusion group, and both drug infusion group. Time-survival estimation curves were generated using Kaplan-Meier analysis. Differences in survival according to intraoperative dopamine infusion were compared using the log-rank test.

## Results

Among the 381 patients who underwent open AAA repair surgery during the study period, 294 patients were included and analyzed (Supplementary Fig. 1 in Additional file 1). The baseline patient characteristics were presented in Table 1 and preoperative data and intraoperative profile were presented in Table 2. Of the 294 patients, postoperative AKI developed in 64 patients (21.8%) after open AAA surgery. Postoperative AKI occurred in 17 out of 44 patients who received an intraoperative dopamine infusion and in 47 out of 250 patients who did not receive an intraoperative dopamine infusion, respectively (38.6 vs. 18.8%, *p* = 0.003, Supplementary Fig. 2 in Additional file 1). In addition, the incidences of AKI were higher in all range groups of dopamine dose than no dopamine group (Supplementary Fig. 3 in Additional file 1). Moreover, the incidence of AKI in patients who received renal-dose dopamine infusion (≤ 3 mcg/kg/min) was higher than patients who did not receive dopamine infusion (50% vs. 18.8%, *p* < 0.001) or patients who received more than renal-dose dopamine infusion (≤ 3 mcg/kg/min vs. > 3 mcg/kg/min; 50% vs. 28.6%, *p* = 0.0081).

**Fig. 1:**
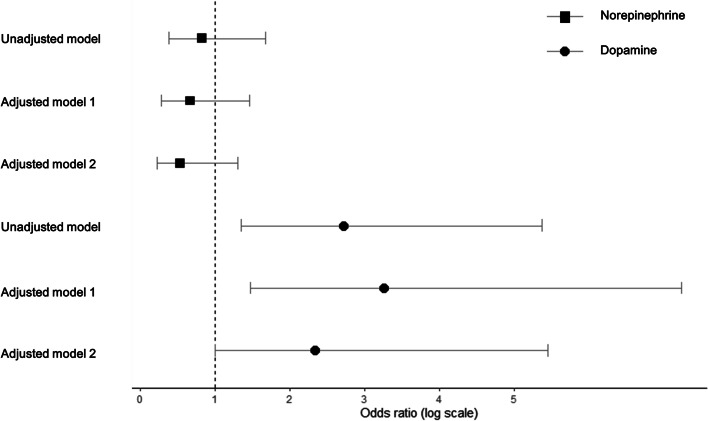
Forest plot showing the risk of AKI of norepinephrine and dopamine infusion in patients undergoing abdominal aortic aneurysm repair before propensity score matching. The squares mark the odds ratio of norepinephrine infusion in each model and the circles mark the odds ratio of dopamine infusion in each model. Horizontal lines show the 95% confidence intervals. *p < 0.01 ^a^Adjusted model 1: adjusted for demographic data, medical history, medication history, and preoperative data. ^b^Adjusted model 2: adjusted for all variables used in adjusted model 1 as well as the operative data listed in Table 1

The results of the logistic regression analysis are summarized in Supplementary Table 1. Dopamine infusion was associated with an increased risk of postoperative AKI in the unadjusted model, adjusted model 1, and adjusted model 2 (unadjusted model, odds ratio [OR] 2.72, 95% confidence interval [CI], 1.35–5.37, *p* = 0.004; adjusted model 1, OR 3.26, 95% CI 1.47–7.23, *p* = 0.003; adjusted model 2, OR 2.34, 95% CI 1.00–5.45, *p* = 0.049; Fig. 1, Supplementary Table 1 in Additional file 1).

In the propensity score-matched samples (44 patients who received the dopamine infusion vs. 132 patients who did not receive the dopamine infusion), the overall incidence of postoperative AKI was 44 out of 176 (25%). The dopamine infusion was an independent risk factor for postoperative AKI in all multivariable analyses in the matched samples (adjusted model 1, OR 2.89, 95% CI 1.27–6.52, *p* = 0.011; adjusted model 2, OR 2.67, 95% CI 1.25–5.70, *p* = 0.030; Supplementary Table 1 in Additional file 1).

In contrast, when the same analyses were done according to the use of norepinephrine, the intraoperative norepinephrine infusion group showed a tendency for a decrease in postoperative AKI (Fig. 1). In addition, the norepinephrine infusion was not associated with postoperative AKI after propensity score matching (adjusted model 1, OR 0.45, 95% CI 0.20–1.03, *p* = 0.058; adjusted model 2, OR 0.64, 95% CI 0.31–1.32, *p* = 0.225; Supplementary Table 2 in Additional file 1) without statistical significance. Furthermore, the patients who received an intraoperative norepinephrine infusion had a lower incidence of postoperative AKI (use vs. no use; 19.3 vs. 22.4%; *p* = 0.615, Supplementary Fig. 2 in Additional file 1).

**Fig. 2:**
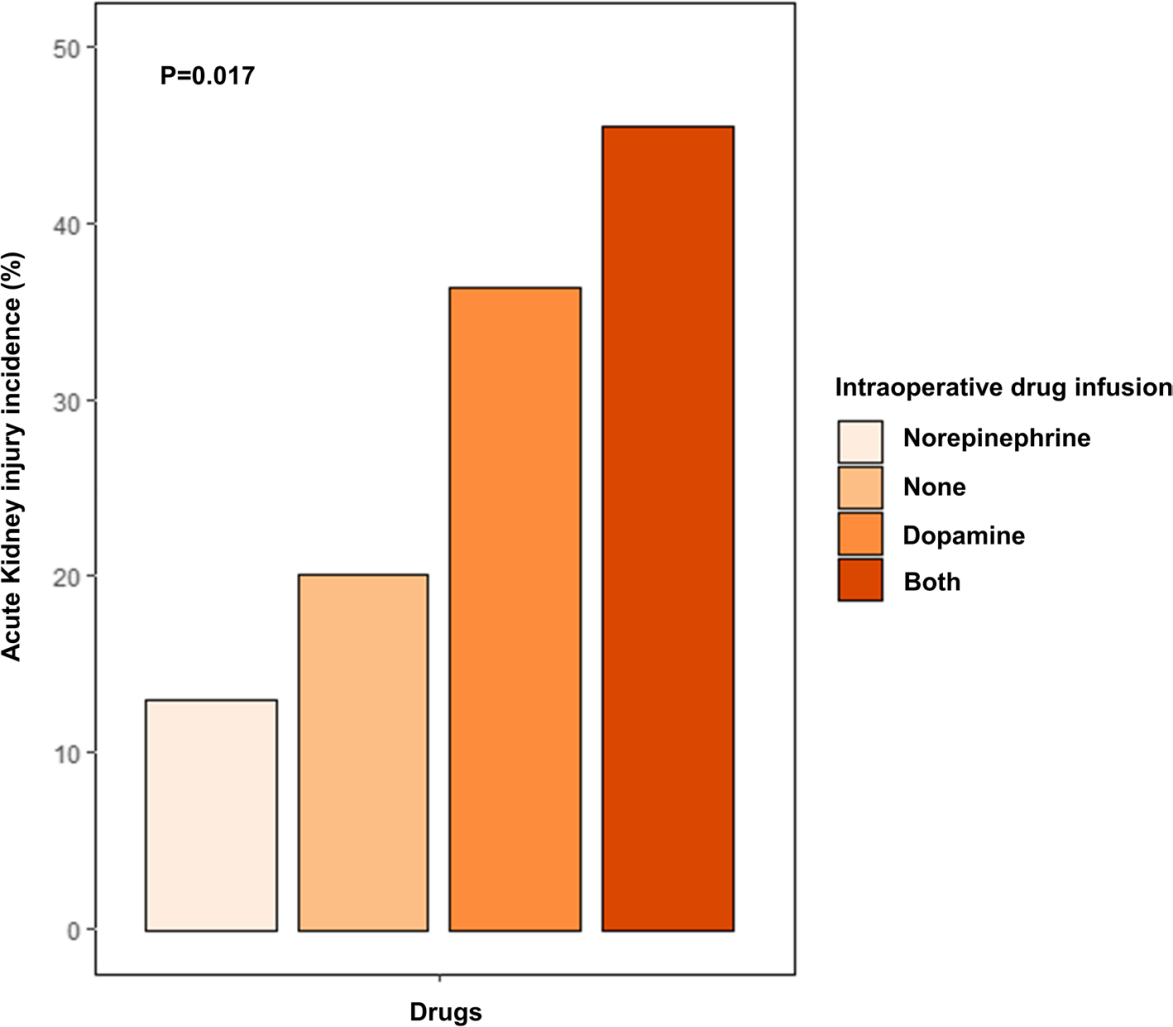
Incidence of acute kidney injury according to the two intraoperative drug use divided into four groups (*n* = 46 for norepinephrine-only group, *n* = 204 for no infusion group, *n* = 33 for dopamine-only infusion group, and *n* = 11 for both drug infusion group). The χ2 test was performed to determine the statistical significance

When the patients were divided into four groups (norepinephrine infusion group, dopamine infusion group, no drug infusion group, and both drug infusion group), the incidence of AKI was highest in both drug infusion group (45.4%) followed by the dopamine infusion group (36.4%) and the no drug infusion group (20.1%). The norepinephrine infusion group (13.0%) had the lowest incidence of postoperative AKI (13.0%) (Fig. 2).

**Fig. 3:**
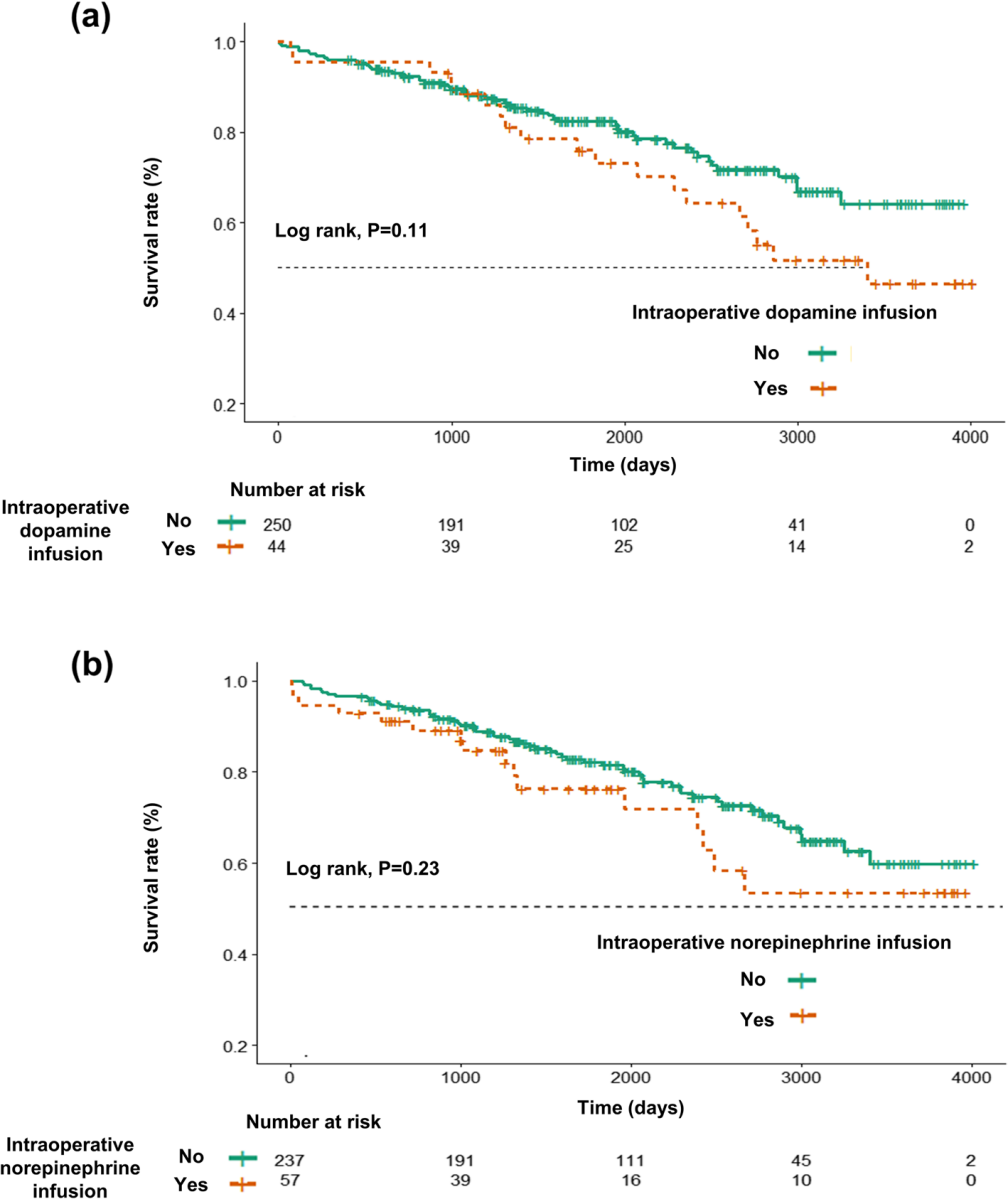
Kaplan-Meier survival curves according to intraoperative dopamine infusion (a) and intraoperative norepinephrine infusion (b) in patients undergoing abdominal aortic aneurysm repair. Censored data are marked with vertical segments

The Kaplan-Meier survival curve showed a tendency for lower survival in both of the dopamine and the norepinephrine infusion group without statistical significance (Fig. 3).

The other secondary outcomes are summarized in Table 3. Patients who received the dopamine infusion underwent postoperative RRT five times more frequently than patients who did not receive the dopamine infusion (6.8 vs. 1.2%; *p* = 0.045). The ICU length of stay was comparable between the two groups (1 vs. 1 day; *p* = 0.996). Hospital length of stay was significantly longer in patients who received an intraoperative dopamine infusion (18 vs. 16 days, *p* = 0.024).

**Table 3:** Postoperative outcome according to intraoperative dopamine infusion

	Dopamine infusion		*P* value
	No (*n =* 250)	Yes (*n =* 44)	
Postoperative RRT	3 (1.2%)	3 (6.8%)	0.045
ICU length of stay	1 [0–2]	1 [0–6]	0.996
Hospital length of stay	16 [13–22]	18 [15–24]	0.024

Data are presented as median [IQR], or number (%)

*ICU* Intensive care unit, *RRT* Renal replacement therapy

## Discussion

In this retrospective study, intraoperative dopamine infusion was associated with more than three times the incidence of postoperative AKI, more than five times postoperative RRT, and a longer hospital length of stay in patients undergoing open AAA surgery. Intraoperative norepinephrine infusion was not associated with postoperative AKI, but rather tended to decrease the incidence of postoperative AKI.

Surgery is a leading cause of AKI in hospitalized patients, accounting for up to 40% of in-hospital AKI cases [20]. Numerous studies have demonstrated that postoperative AKI in patients undergoing noncardiac surgery is associated with high in-hospital mortality, long-term mortality, and an increase in cardiovascular complications [21]. Even in patients undergoing cardiac surgery, postoperative AKI is a well-known complication that results in higher adverse postoperative outcomes, including a high 90-day mortality rate and increased hospital costs. The mechanisms of postoperative renal dysfunction are multifactorial and are associated with renal ischemia, circulatory failure, and nephrotoxic agents as well as an inflammatory stress response to surgery [22, 23].

AAA repair is one of most high-risk noncardiac surgeries for postoperative AKI. Up to 33% of patients undergoing AAA repair develop postoperative AKI [5–9]. The development of AKI is known to affect postoperative morbidity, mortality, and length of stay in patients undergoing AAA surgery [12, 24, 25]. Patients requiring RRT after AAA surgery have a high mortality rate [26, 27]. Additionally, postoperative AKI not requiring RRT also increases the risk of postoperative mortality in patients undergoing open AAA repair [28].

Therefore, it is important to identify the risk factors that affect the development of AKI after AAA surgery and correct the adjustable factors. However, research is insufficient on whether any intraoperative intravenous infusion drug can affect postoperative renal outcomes after AAA repair.

Dopamine is an endogenous catecholamine that influences different catecholamine receptors depending on the dose [29, 30]. Low-dose dopamine has been used in intensive care patients for its reno-protective effects [31]. Low-dose dopamine is associated with increased renal blood flow and diuresis by renal vasodilation, which may explain the mechanism of the effect of dopamine on preventing AKI [15, 31, 32]. In addition, low-dose dopamine is presumed to protect the kidneys by minimizing endogenous norepinephrine-induced vasoconstriction mediated by the stimulation of dopamine-1 receptors [33, 34].

However, several clinical studies have reported controversial results on the reno-protective effects of dopamine [15–17, 35, 36]. A prospective, randomized controlled trial demonstrated that a low dose of dopamine infusion does not prevent the development of AKI, nor does it improve outcomes in patients undergoing cardiac surgery [16]. Another randomized controlled trial of 52 patients undergoing elective coronary artery bypass grafting surgery showed that a dopamine infusion beginning at the induction of anesthesia and continued for 24 h was not associated with improved renal function [37]. Furthermore, Lauschke et al. [17] suggested that low-dose dopamine could worsen renal perfusion in patients with AKI.

Several studies have attempted to explain the mechanism of the dopamine effects on the development of AKI. Dopamine is presumed to have a diuretic effect on the proximal tubules, increasing the reabsorption of chloride at the ascending limb of the loop of Henle, which may increase medullary oxygen consumption and exacerbate medullary ischemia [38]. Schenarts et al. [39] proposed that dopamine appears to damage renal oxygen kinetics and defects in feedback systems that protect the kidney from ischemia leading to the development of AKI [39]. In addition, previous research has shown that dopamine potentially causes or worsens hypovolemia and prerenal acute renal failure through its natriuretic effects and may induce hypokalemia and hypophosphatemia in critically ill patients [17]. These reports provide evidence that supports the rationale for reconsidering the routine use of low-dose dopamine in critically ill patients.

There has been insufficient studies which evaluated the effect of intraoperative dopamine use on the postoperative AKI in patients undergoing AAA. A randomized controlled trial with a small number of patients (*n* = 37) undergoing elective major abdominal vascular surgery suggested that a low-dose dopamine infusion during the postoperative period failed to show protective reno-protective effect [35]. In a retrospective study on 162 AAA repair patients, an intraoperative dopamine infusion has been associated with postoperative complications. Although those postoperative complications included renal complications, the separate analysis data on AKI was not provided [40].

Although the association was not significant, the norepinephrine infusion appeared to be associated with a decreased incidence of AKI, suggesting that norepinephrine successfully maintained renal perfusion without damaging the kidneys. The previous study has proven that norepinephrine increased cardiac index and improved systemic oxygen delivery dose-dependently via the beta 1 effect [41]. In addition, norepinephrine induces an increase in renal perfusion pressure by increasing renal vascular resistance via vasoconstriction mediated by the alpha-1 effect and pressure-dependent renal auto-regulation [42]. Therefore, norepinephrine may be an alternative to dopamine. It would be valuable to validate this finding in a prospective study with a large number of patients.

Several limitations of this study should be discussed. First, the data of total amount of dopamine infused and the duration might not be accurate and were partially missing. This was due to manual recording of the dopamine doses, which is often insufficient and missing to precisely calculate the total dose of the dopamine infusion. However, when comparisons of the incidence of AKI after AAA depending on dopamine doses were performed using available data, the study showed similar result. Second, the total number of subjects analyzed was small and the number of representatives in each group was also small before and after matching. Nevertheless, statistical significance was found. Therefore, we may need a large-scale randomized prospective study to validate our results. In addition, as we excluded patients with prior diagnosis of chronic kidney disease, current results cannot be applied to those patients. Third, the urine output criterion was not used for the AKI diagnosis. This is a retrospective study and in our hospital, urine output records has been recorded manually and often missing, resulting in inaccurate values to be analyzed. However, serum creatinine can be a sensitive marker for AKI as small changes in creatinine levels have shown to be associated with comorbidities and mortality after surgery [43]. Fourth, other crucial factors such as clamping time are missing. In addition to the shortcomings inherent to retrospective studies, missing value, which result from absence of records could not be included. However, the duration of surgery we used in this study correlates with the clamping time and therefore may represent the clamping time. Fifth, we analyzed all eligible AAA surgery that took place in recent 9 years in our institution, though there might have been considerable changes in surgical technique and/or anesthetic management. We adjusted the ‘surgery year’ to consider potential effect of development of surgical technique and/or anesthetic management on the outcome. Sixth, the survival analysis was not adjusted for other factors that may have affected the survival outcome. Therefore, the tendency in the survival differences need to be interpreted with caution. Seventh, it seems the dopamine group received more total colloid volume and pack RBC units, which are known to cause AKI and may act as a confounding factor. However, we performed multivariate analysis adjusting for variables that may affect AKI including the total amount of colloid volume and transfusion. Therefore, we believe that our study highlighted the effects of dopamine on AKI after AAA. Lastly, due to the retrospective nature of this study, there could be hidden or unmeasurable confounding factors such as physician’s preference in which drug is chosen for infusion. Further prospective trials would be needed to confirm the results of current study.

## Conclusions

In conclusion, intraoperative dopamine use greatly increased postoperative AKI and was associated with higher postoperative RRT and a longer hospital length of stay in patients undergoing AAA repair. However, intraoperative norepinephrine infusion did not increase the incidence of AKI. The use of intraoperative dopamine should be avoided during open AAA repair. We may be careful to use dopamine during open AAA repair.

## Supplementary Information

**Additional file 1.** Supplementary figure and tables

## Data Availability

The datasets used and/or analyzed during the current study are available from the corresponding author on reasonable request.

## Abbreviations

AKI: Acute kidney injury
AAA: Abdominal aortic aneurysm
RRT: Renal replacement therapy
OR: Odd ratio
CI: Confidence interval
Scr: Serum creatinine
KDIGO: Kidney Disease Improving Global Outcomes
IRB: Institutional Review Board
ICU: intensive care unit
GEE: generalised estimating equation
ACEi: angiotensin converting enzyme inhibitor
ARB: angiotensin II receptor blocker
RBC: red blood cell
