# Supplementary material for: Relationship between intraoperative dopamine infusion and postoperative acute kidney injury in patients undergoing open abdominal aorta aneurysm repair
Source: BMC Anesthesiol. 2022 Mar 26;22:82. doi: 10.1186/s12871-022-01624-6 (PMC8962567; doi:10.1186/s12871-022-01624-6)
Supplement: Supplementary file 1 — Additional file 1. Supplementary figure and tables [file 12871_2022_1624_MOESM1_ESM.docx]

**Appendix A. Additional files**

**Supplementary Figures**


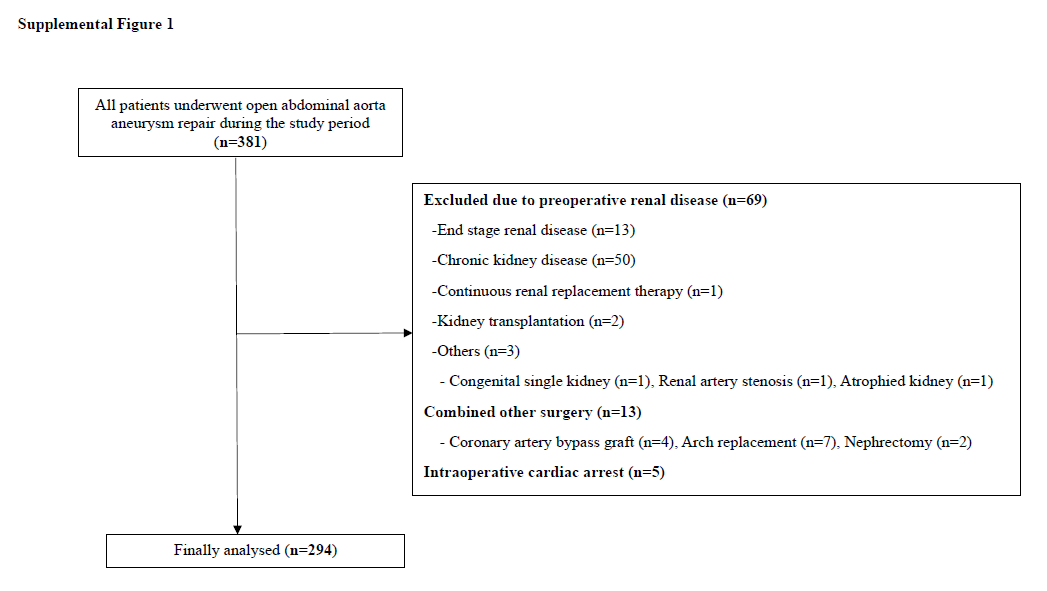


**Supplemental Figure 1.** Study flowchart for patient inclusion.


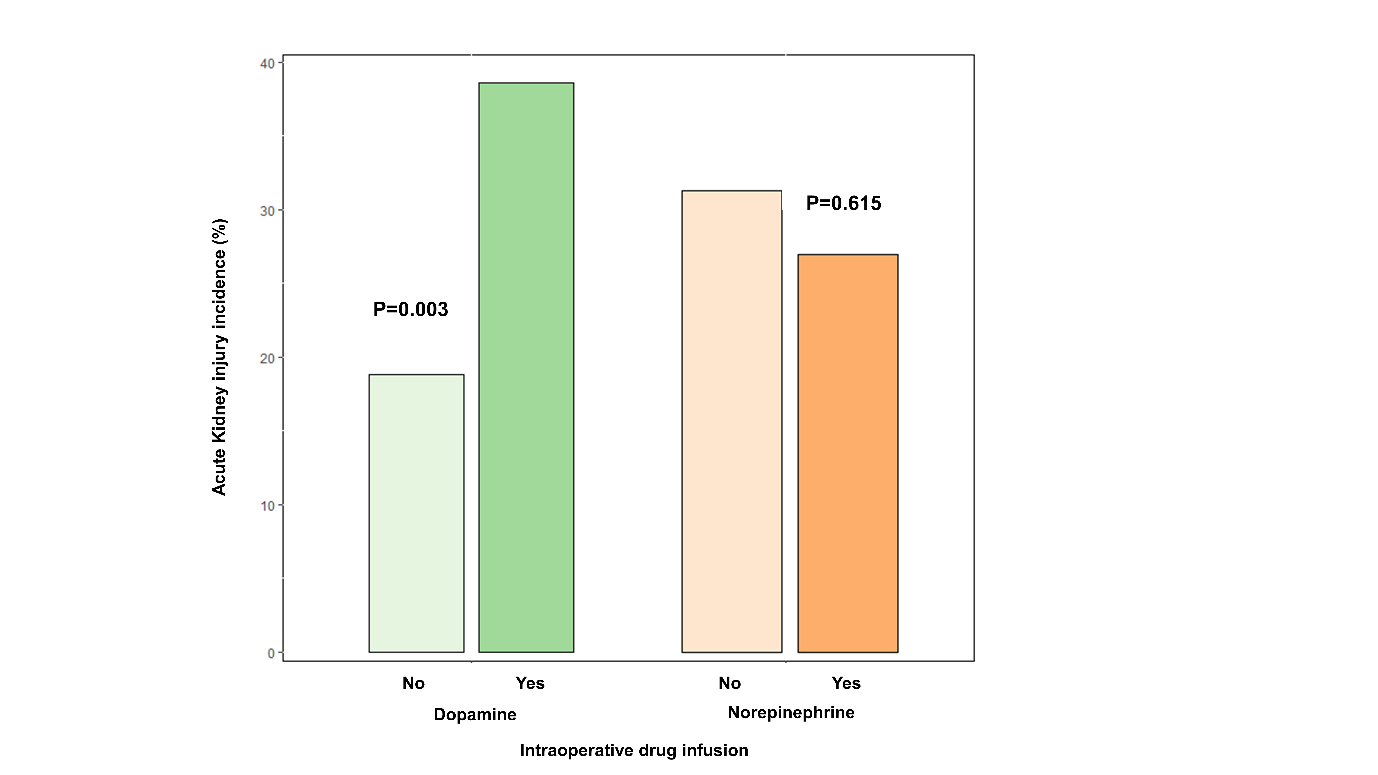


**Supplemental Figure 2.** Incidence of acute kidney injury according to intraoperative dopamine (n=44 out of 294) or norepinephrine (n=57 out of 294) use. The χ^2^ test was performed to determine the statistical significance.


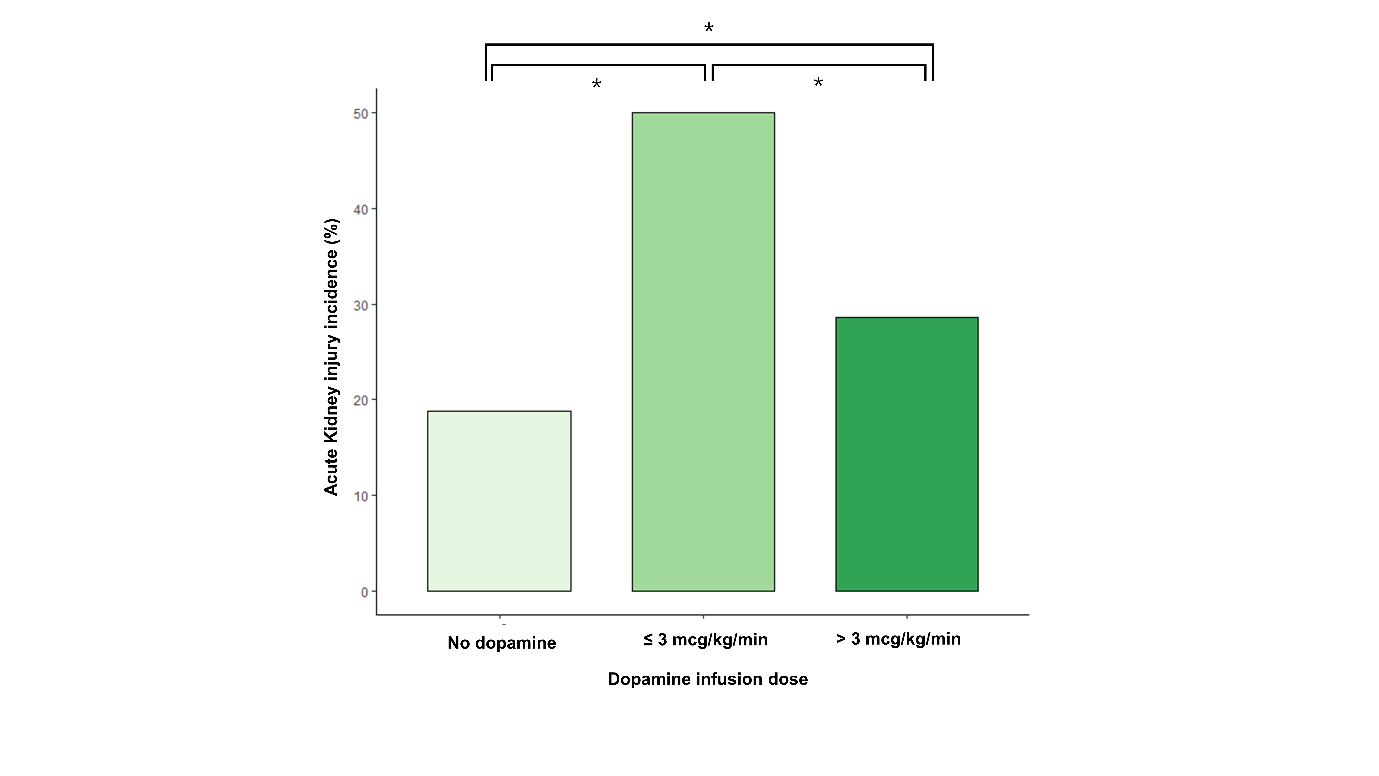


**Supplementary Figure 3.** Incidence of AKI according to the dose of dopamine infusion. Postoperative AKI occurred in 5 out of 10 patients who received renal-dose dopamine infusion (≤ 3 mcg/kg/min) and in 8 out of 28 patients who received more than renal-dose dopamine infusion, respectively (≤ 3 mcg/kg/min vs. > 3 mcg/kg/min; 50% vs. 28.6%, *p*=0.0081). The number of patients whom the dose of dopamine were missing was 6. The χ2 test with Bonferroni correction was performed to determine the statistical significance. All comparisons were statistically significant marked with asterisk (*)

**Supplementary Tables**

**Supplementary Table 1**. Multivariable regression analysis and multivariable generalized estimating equation analysis after propensity score matching for postoperative acute kidney injury after intraoperative dopamine infusion in patients undergoing aortic aneurysm.

|  | Unadjusted model | |  | Adjusted model 1* | |  | Adjusted model 2† | |
| --- | --- | --- | --- | --- | --- | --- | --- | --- |
|  | OR(95% CI) | P value |  | OR(95% CI) | P value |  | OR(95% CI) | P value |
| **Before matching** |  |  |  |  |  |  |  |  |
| Dopamine continuous infusion |  |  |  |  |  |  |  |  |
| No | ref |  |  | ref |  |  | ref |  |
| Yes | 2.72 (1.35-5.37) | 0.004 |  | 3.26 (1.47-7.23) | 0.003 |  | 2.34 (1.00-5.45) | 0.049 |
| Demographic data |  |  |  |  |  |  |  |  |
| Age (year) | 1.03 (1.00-1.07) | 0.053 |  | 1.02 (0.98-1.06) | 0.288 |  | 1.02 (0.98-1.06) | 0.353 |
| Female | 0.94 (0.47-1.99) | 0.865 |  | 0.81 (0.34-1.84) | 0.624 |  | 0.78 (0.34-1.82) | 0.568 |
| Body mass index |  |  |  |  |  |  |  |  |
| < 18.5 kg/m^2^ | Reference |  |  |  |  |  |  |  |
| 18.5–24.9 kg/m^2^ | 0.45 (0.17-1.27) | 0.113 |  |  |  |  |  |  |
| 25.0–29.9 kg/m^2^ | 0.57 (0.20-1.70) | 0.295 |  |  |  |  |  |  |
| ≥ 30.0 kg/m^2^ | 0.51 (0.09-2.32) | 0.397 |  |  |  |  |  |  |
| Past medical history |  |  |  |  |  |  |  |  |
| Hypertension | 2.02 (1.08-3.99) | 0.035 |  | 1.04 (0.46-2.42) | 0.920 |  | 1.11 (0.49-2.56) | 0.800 |
| Diabetes mellitus | 2.11 (1.01-4.26) | 0.042 |  | 1.63 (0.70-3.67) | 0.246 |  | 1.28 (0.55-2.99) | 0.563 |
| Dyslipidemia | 0.96 (0.52-1.72) | 0.889 |  |  |  |  |  |  |
| Chronic liver disease | 1.03 (0.15-4.38) | 0.973 |  |  |  |  |  |  |
| Cerebrovascular disease | 2.05 (0.74-5.25) | 0.144 |  | 2.41 (0.73-7.46) | 0.134 |  | 2.20 (0.68-7.14) | 0.191 |
| Angina pectoris | 2.28 (1.18-4.30) | 0.012 |  | 2.19 (0.96-4.93) | 0.059 |  | 2.79 (1.19-6.59) | 0.019 |
| Myocardial infarction | 1.76 (0.65-4.39) | 0.240 |  |  |  |  |  |  |
| Atrial fibrillation | 1.84 (0.38-7.17) | 0.400 |  |  |  |  |  |  |
| Chronic obstructive pulmonary disease | 1.06 (0.34-2.81) | 0.910 |  |  |  |  |  |  |
| Medication history |  |  |  |  |  |  |  |  |
| Aspirin | 1.23 (0.63-2.32) | 0.529 |  |  |  |  |  |  |
| Clopidogrel | 1.61 (0.79-3.19) | 0.177 |  | 1.35 (0.55-3.17) | 0.501 |  | 1.15 (0.46-2.82) | 0.768 |
| ACEi/ARB | 1.58 (0.89-2.77) | 0.115 |  | 1.65 (0.82-3.36) | 0.162 |  | 1.51 (0.73-3.10) | 0.263 |
| ß blocker | 1.29 (0.69-2.34) | 0.412 |  |  |  |  |  |  |
| Calcium channel blocker | 1.80 (1.02-3.15) | 0.041 |  | 1.80 (0.92-3.53) | 0.086 |  | 1.63 (0.82-3.25) | 0.162 |
| Diuretics | 2.62 (1.28-5.22) | 0.007 |  | 2.03 (0.91-4.46) | 0.079 |  | 2.12 (0.93-4.81) | 0.073 |
| Statin | 0.75 (0.42-1.32) | 0.328 |  |  |  |  |  |  |
| Oral hypoglycemic agents | 1.94 (0.83-4.31) | 0.110 |  |  |  |  |  |  |
| Preoperative data |  |  |  |  |  |  |  |  |
| Serum creatinine (mg/dl) | 1.21 (0.45-3.16) | 0.697 |  |  |  |  |  |  |
| Hematocrit (%) | 0.94 (0.89-0.99) | 0.014 |  | 0.95 (0.89-1.01) | 0.129 |  | 0.94 (0.88-1.01) | 0.091 |
| Suprarenal (%) | 3.77 (1.56-9.07) | 0.003 |  | 4.96 (1.86-13.38) | 0.001 |  | 3.80 (1.26-11.43) | 0.018 |
| Diameter(mm) | 1.02 (1.01-1.04) | 0.010 |  | 1.02 (1.00-1.04) | 0.119 |  | 1.01 (0.99-1.03) | 0.277 |
| Operation data |  |  |  |  |  |  |  |  |
| Emergency surgery | 0.99 (0.40-2.20) | 0.981 |  |  |  |  |  |  |
| Duration of surgery (min) | 1.00 (1.00-1.01) | 0.027 |  |  |  |  | 1.00 (1.00-1.00) | 0.825 |
| Lowest MAP | 0.96 (0.93-0.99) | 0.041 |  |  |  |  | 0.97 (0.93-1.01) | 0.140 |
| Nitroglycerin use | 1.49 (0.76-2.84) | 0.233 |  |  |  |  |  |  |
| Mannitol use | 2.77 (1.50-5.07) | < 0.001 |  |  |  |  | 2.22 (1.04-4.73) | 0.039 |
| Furosemide use | 2.70 (1.54-4.79) | < 0.001 |  |  |  |  | 1.78 (0.87-3.64) | 0.117 |
| Hydroxyethyl starch use | 1.41 (0.79-2.60) | 0.250 |  |  |  |  |  |  |
| RBC transfusion (Units) | 1.006 (0.99-1.03) | 0.531 |  |  |  |  |  |  |
| Surgery year | 0.94 (0.85-1.04) | 0.241 |  |  |  |  |  |  |
|  |  |  |  |  |  |  |  |  |
| **After matching** |  |  |  |  |  |  |  |  |
| Dopamine continuous infusion |  |  |  |  |  |  |  |  |
| No | ref |  |  | ref |  |  | ref |  |
| Yes | 2.34 (1.12-4.87) | 0.023 |  | 2.89 (1.27-6.52) | 0.011 |  | 2.67 (1.25-5.70) | 0.011 |

Propensity score matching used all variables listed in supplementary Table 1 (Adjusted model 1) except OHA due to multi-collinearity.

In the matching procedure, the nearest neighbor-matching method with 1:3 pairing was used without the due to the reduced number of data in 1:1 matching

OR, odds ratio; CI, confidence interval.; ACEi, angiotensin converting enzyme inhibitor; ARB, angiotensin II receptor blocker; RBC, red blood cell; MAP, mean arterial blood pressure.

*Multivariable model 1 was adjusted for demographic data, past medical history, medication history, preoperative data.

† Multivariable model 2 was adjusted for all variables used in Multivariable model 1 and operative data listed in Table 1.

**Supplementary Table 2**. Multivariable regression analysis and multivariable generalized estimating equation analysis after propensity score matching for postoperative acute kidney injury after intraoperative norepinephrine infusion in patients undergoing aortic aneurysm.

|  | Unadjusted model | |  | Adjusted model 1* | |  | Adjusted model 2† | |
| --- | --- | --- | --- | --- | --- | --- | --- | --- |
|  | OR(95% CI) | P value |  | OR(95% CI) | P value |  | OR(95% CI) | P value |
| **Before matching** |  |  |  |  |  |  |  |  |
| Norepinephrine continuous infusion |  |  |  |  |  |  |  |  |
| No | ref |  |  | ref |  |  | ref |  |
| Yes | 0.83 (0.39-1.67) | 0.615 |  | 0.67 (0.28-1.46) | 0.331 |  | 0.54 (0.23-1.31) | 0.173 |
| Demographic data |  |  |  |  |  |  |  |  |
| Age (year) | 1.03 (1.00-1.07) | 0.053 |  | 1.02 (0.99-1.06) | 0.239 |  | 1.02 (0.98-1.06) | 0.345 |
| Female | 0.94 (0.47-1.99) | 0.865 |  | 0.73 (0.30-1.66) | 0.459 |  | 0.73 (0.31-1.72) | 0.471 |
| Body mass index |  |  |  |  |  |  |  |  |
| < 18.5 kg m^-2^ | Reference |  |  |  |  |  |  |  |
| 18.5–24.9 kg m^-2^ | 0.45 (0.17-1.27) | 0.113 |  |  |  |  |  |  |
| 25.0–29.9 kg m^-2^ | 0.57 (0.20-1.70) | 0.295 |  |  |  |  |  |  |
| ≥ 30.0 kg m^-2^ | 0.51 (0.09-2.32) | 0.397 |  |  |  |  |  |  |
| Past medical history |  |  |  |  |  |  |  |  |
| Hypertension | 2.02 (1.08-3.99) | 0.035 |  | 1.02 (0.46-2.33) | 0.957 |  | 1.12 (0.49-2.56) | 0.780 |
| Diabetes mellitus | 2.11 (1.01-4.26) | 0.042 |  | 1.57 (0.68-3.48) | 0.281 |  | 1.24 (0.54-2.85) | 0.620 |
| Dyslipidemia | 0.96 (0.52-1.72) | 0.889 |  |  |  |  |  |  |
| Chronic liver disease | 1.03 (0.15-4.38) | 0.973 |  |  |  |  |  |  |
| Cerebrovascular disease | 2.05 (0.74-5.25) | 0.144 |  | 2.06 (0.66-6.07) | 0.198 |  | 1.97 (0.63-6.20) | 0.247 |
| Angina pectoris | 2.28 (1.18-4.30) | 0.012 |  | 2.10 (0.95-4.6) | 0.065 |  | 2.98 (1.27-6.99) | 0.012 |
| Myocardial infarction | 1.76 (0.65-4.39) | 0.240 |  |  |  |  |  |  |
| Atrial fibrillation | 1.84 (0.38-7.17) | 0.400 |  |  |  |  |  |  |
| Chronic obstructive pulmonary disease | 1.06 (0.34-2.81) | 0.910 |  |  |  |  |  |  |
| Medication history |  |  |  |  |  |  |  |  |
| Aspirin | 1.23 (0.63-2.32) | 0.529 |  |  |  |  |  |  |
| Clopidogrel | 1.61 (0.79-3.19) | 0.177 |  | 1.28 (0.53-2.97) | 0.565 |  | 1.05 (0.43-2.57) | 0.921 |
| ACEi/ARB | 1.58 (0.89-2.77) | 0.115 |  | 1.44 (0.73-2.86) | 0.294 |  | 1.36 (0.67-2.74) | 0.398 |
| ß blocker | 1.29 (0.69-2.34) | 0.412 |  |  |  |  |  |  |
| Calcium channel blocker | 1.80 (1.02-3.15) | 0.041 |  | 1.91 (0.99-3.72) | 0.055 |  | 1.67 (0.84-3.31) | 0.143 |
| Diuretics | 2.62 (1.28-5.22) | 0.007 |  | 1.91 (0.85-4.22) | 0.111 |  | 1.94 (0.84-4.48) | 0.121 |
| Statin | 0.75 (0.42-1.32) | 0.328 |  |  |  |  |  |  |
| Oral hypoglycemic agents | 1.94 (0.83-4.31) | 0.110 |  |  |  |  |  |  |
| Preoperative data |  |  |  |  |  |  |  |  |
| Serum creatinine (mg dl^-1^) | 1.21 (0.45-3.16) | 0.697 |  |  |  |  |  |  |
| Hematocrit (%) | 0.94 (0.89-0.99) | 0.014 |  | 0.95 (0.89-1.02) | 0.131 |  | 0.94 (0.88-1.01) | 0.103 |
| Suprarenal (%) | 3.77 (1.56-9.07) | 0.003 |  | 4.33 (1.67-11.25) | 0.002 |  | 3.25 (1.11-9.55) | 0.032 |
| Diameter(mm) | 1.02 (1.01-1.04) | 0.010 |  | 1.02 (1.01-1.04) | 0.031 |  | 1.02 (1.00-1.04) | 0.139 |
| Operation data |  |  |  |  |  |  |  |  |
| Emergency surgery | 0.99 (0.40-2.20) | 0.981 |  |  |  |  |  |  |
| Duration of surgery (min) | 1.00 (1.00-1.01) | 0.027 |  |  |  |  | 1.00 (1.00-1.00) | 0.920 |
| Lowest MAP | 0.96 (0.93-0.99) | 0.041 |  |  |  |  | 0.96 (0.92-1.00) | 0.054 |
| Nitroglycerin use | 1.49 (0.76-2.84) | 0.233 |  |  |  |  |  |  |
| Mannitol use | 2.77 (1.50-5.07) | < 0.001 |  |  |  |  | 2.36 (1,12-4.97) | 0.024 |
| Furosemide use | 2.70 (1.54-4.79) | < 0.001 |  |  |  |  | 2.19 (1.08-4.43) | 0.030 |
| Hydroxyethyl starch use | 1.41 (0.79-2.60) | 0.250 |  |  |  |  |  |  |
| RBC transfusion (Units) | 1.006 (0.99-1.03) | 0.531 |  |  |  |  |  |  |
| Surgery year | 0.94 (0.85-1.04) | 0.241 |  |  |  |  |  |  |
|  |  |  |  |  |  |  |  |  |
| **After matching** |  |  |  |  |  |  |  |  |
| Norepinephrine continuous infusion |  |  |  |  |  |  |  |  |
| No | ref |  |  | ref |  |  | ref |  |
| Yes | 0.81 (0.41-1.6) | 0.543 |  | 0.45 (0.20-1.03) | 0.058 |  | 0.64 (0.31-1.32) | 0.225 |

Propensity score matching used all variables listed in supplementary Table 2(Adjusted model 1) except OHA due to multi-collinearity.

In the matching procedure, the nearest neighbor-matching method with 1:3 pairing was used without the due to the reduced number of data in 1:1 matching

OR, odds ratio; CI, confidence interval.; ACEi, angiotensin converting enzyme inhibitor; ARB, angiotensin II receptor blocker; RBC, red blood cell; MAP, mean arterial blood pressure.

*Multivariable model 1 was adjusted for demographic data, past medical history, medication history, preoperative data..

† Multivariable model 2 was adjusted for all variables used in Multivariable model 1 and operative data listed in Table 1.
